# Supplementary material for: Modeling glioblastoma heterogeneity as a dynamic network of cell states
Source: Mol Syst Biol. 2021 Sep 16;17(9):e10105. doi: 10.15252/msb.202010105 (PMC8444284; doi:10.15252/msb.202010105)
Supplement: Supplementary file 5 — Source Data for Figure 3 [file MSB-17-e10105-s001.zip › Figure3A_sourcedata/GSEA_3065/nosigdb_state4.GseaPreranked.1622718430994/SUVA_AC_PMID_31327527.html]

Details for gene set SUVA\_AC\_PMID\_31327527[GSEA]

|  || Dataset | state4 |
| Phenotype | NoPhenotypeAvailable |
| Upregulated in class | na\_pos |
| GeneSet | SUVA\_AC\_PMID\_31327527 |
| Enrichment Score (ES) | 0.6991951 |
| Normalized Enrichment Score (NES) | 2.3440576 |
| Nominal p-value | 0.0 |
| FDR q-value | 0.0 |
| FWER p-Value | 0.0 |
Table: GSEA Results Summary

  

Fig 1: Enrichment plot: SUVA\_AC\_PMID\_31327527      
 Profile of the Running ES Score & Positions of GeneSet Members on the Rank Ordered List

  

| PROBE | GENE SYMBOL | GENE\_TITLE | RANK IN GENE LIST | RANK METRIC SCORE | RUNNING ES | CORE ENRICHMENT || 1 | GFAP |  |  | 0 | 0.995 | 0.1213 | Yes |
| 2 | PMP2 |  |  | 3 | 0.727 | 0.2095 | Yes |
| 3 | FABP7 |  |  | 5 | 0.656 | 0.2893 | Yes |
| 4 | S100B |  |  | 6 | 0.630 | 0.3661 | Yes |
| 5 | SPARCL1 |  |  | 17 | 0.416 | 0.4151 | Yes |
| 6 | RAB31 |  |  | 21 | 0.388 | 0.4619 | Yes |
| 7 | HOPX |  |  | 28 | 0.360 | 0.5048 | Yes |
| 8 | MT3 |  |  | 38 | 0.318 | 0.5420 | Yes |
| 9 | HEY1 |  |  | 39 | 0.318 | 0.5807 | Yes |
| 10 | CLU |  |  | 95 | 0.252 | 0.6025 | Yes |
| 11 | TTYH1 |  |  | 100 | 0.249 | 0.6322 | Yes |
| 12 | GATM |  |  | 156 | 0.219 | 0.6501 | Yes |
| 13 | ATP1B2 |  |  | 159 | 0.217 | 0.6762 | Yes |
| 14 | EDNRB |  |  | 271 | 0.180 | 0.6804 | Yes |
| 15 | GPM6B |  |  | 346 | 0.166 | 0.6887 | Yes |
| 16 | SPARC |  |  | 539 | 0.132 | 0.6740 | Yes |
| 17 | MLC1 |  |  | 594 | 0.126 | 0.6807 | Yes |
| 18 | PLTP |  |  | 625 | 0.123 | 0.6908 | Yes |
| 19 | PMP22 |  |  | 765 | 0.107 | 0.6815 | Yes |
| 20 | CST3 |  |  | 792 | 0.104 | 0.6901 | Yes |
| 21 | ANXA5 |  |  | 814 | 0.103 | 0.6992 | Yes |
| 22 | SLC1A3 |  |  | 1069 | 0.083 | 0.6686 | No |
| 23 | AGT |  |  | 1103 | 0.082 | 0.6733 | No |
| 24 | RAMP1 |  |  | 1283 | 0.072 | 0.6532 | No |
| 25 | HEPN1 |  |  | 1554 | 0.060 | 0.6172 | No |
| 26 | PRCP |  |  | 1675 | 0.056 | 0.6048 | No |
| 27 | F3 |  |  | 3133 | 0.022 | 0.3736 | No |
| 28 | DBI |  |  | 4122 | -0.017 | 0.2171 | No |
| 29 | S100A16 |  |  | 4787 | -0.067 | 0.1186 | No |
| 30 | PCDHGC3 |  |  | 4939 | -0.078 | 0.1039 | No |
| 31 | PON2 |  |  | 5246 | -0.105 | 0.0676 | No |
| 32 | METTL7B |  |  | 5354 | -0.114 | 0.0643 | No |
| 33 | PTPRZ1 |  |  | 5811 | -0.186 | 0.0138 | No |
| 34 | BCAN |  |  | 6211 | -0.481 | 0.0083 | No |
Table: GSEA details [plain text format]

  

Fig 2: SUVA\_AC\_PMID\_31327527: Random ES distribution      
 Gene set null distribution of ES for **SUVA\_AC\_PMID\_31327527**
